# Experimental measurements of the viscosity and melt structure of alkali basalts at high pressure and temperature

**DOI:** 10.1038/s41598-022-06551-7

**Published:** 2022-02-16

**Authors:** Barbara Bonechi, Vincenzo Stagno, Yoshio Kono, Rostislav Hrubiak, Luca Ziberna, Giovanni B. Andreozzi, Cristina Perinelli, Mario Gaeta

**Affiliations:** 1 Dipartimento di Scienze della Terra, Sapienza Università di Roma, P.le Aldo Moro, 5, 00185 Rome, Italy; 2 Istituto Nazionale di Geofisica e Vulcanologia, 00143 Rome, Italy; 3 Geodynamics Research Center, Ehime University, Matsuyama, 790-8577 Japan; 4 High Pressure Collaborative Access Team, X-Ray Science Division, Argonne National Laboratory, 9700 S. Cass Avenue, Argonne, IL 60439 USA; 5 Dipartimento di Matematica e Geoscienze, Università degli Studi di Trieste, Via Weiss 8, 34128 Trieste, Italy; 6 Bayerisches Geoinstitut, University of Bayreuth, 95440 Bayreuth, Germany

**Keywords:** Solid Earth sciences, Mineralogy, Petrology, Volcanology

## Abstract

Volcanic eruptions are shallow phenomena that represent the final stage of density- and viscosity- driven processes of melt migration from source rocks at upper mantle depths. In this experimental study, we investigated the effect of pressure (0.7–7.0 GPa) and temperature (1335–2000 °C) on the viscosity and the atomic melt structure of a synthetic anhydrous primitive alkaline basalt, an analogue of the pre-eruptive magma that likely feeds the Campi Flegrei Volcanic District at present day. Obtained viscosities (0.5–3.0 Pa s), mobility (0.1–0.4 g cm^3^ Pa^−1^ s^−1^) and ascent velocity (1.5–6.0 m yr^−1^) are presented to support geochemical and geophysical observations of Campi Flegrei as a critical volcanic district currently undergoing gradual magma recharge at depth.

## Introduction

Understanding the rheological properties of magmas at pressures and temperature at which they form is necessary to model the ascent rate from Earth’s mantle up to the surface. Several studies have investigated the viscosity of natural^1,2^ and synthetic magmas at various pressure conditions from atmospheric to ~ 400 MPa^3,4^. Additional studies have provided empirical numerical models to determine the viscosity of melt compositions spanning terrestrial volcanic rocks from basic to silicic, from subalkaline to peralkaline, and from metaluminous to peraluminous^5^ (and references therein). These viscosity models can be safely used to model the viscosity at atmospheric conditions (e.g., lava emplacement) or for shallow magma bodies or conduits where the effect of pressure can be considered negligible. These models cannot be used to predict viscosity at mantle depths where primitive magmas originate and, therefore, the effect of pressure is such to influence their atomic structure^6^. More recent experimental studies have used in-situ X-ray radiography techniques combined with high-pressure apparatus, which have allowed to explore a wide range of simplified silicate melt compositions like peridotitic^7^, basaltic^8^, trachy-andesitic^9^, albitic^10^ and dacitic^11^ melts at high pressure and temperature representative of Earth’s mantle regions. Although such studies highlighted differences in viscosity up to four orders of magnitude within ~ 1.5–13.0 GPa and temperatures up to ~ 2200 °C for melts that have SiO_2_ contents between 46.6 and 67.5 wt%^12^, at present none of these compositions can be taken as unique reference values to model the rheological behavior of mantle-derived subduction-related basalts.

We experimentally determined the viscosity of a primitive alkali basalt composition at pressures (P) representative of upper mantle to reveal the effect of its mobility and ascent velocity on the timing of magmatic supply of deep portions of volcanic plumbing systems where, in absence of faults and dikes, the magma moves by porous flow. Among the diverse suite of basalts, the primitive magma at Campi Flegrei is of interest as it shows geochemical and petrological evidences of a mantle source that might still feed the current pre-eruptive system^13,14^ and lead to dramatic eruptions. With respect to the synthetic simplified tholeiitic composition used by Sakamaki et al*.*^8^, Agee et al*.*^15^ and Ohtani and Maeda^16^, taken as representative of mid-ocean ridge (MOR) basalts, the primitive magma at Campi Flegrei is characterized by lower SiO_2_ content (~ 49 wt%), slightly higher MgO (~ 9 wt%), and almost twice the alkali content (4.46 wt% of Na_2_O + K_2_O) (see Fig. S1 and Table S1 of the Supplementary Materials). To shed light on the mobility and ascent velocity of deep portion of volcanic plumbing systems, a synthetic alkaline basaltic glass (APR16 rock sample^14,17–22^) was prepared. The viscosity was measured in-situ at P of 0.7–7.0 GPa and superliquidus temperatures (1335 °C ≤ T ≤ 2000 °C) using the falling sphere technique with the Paris-Edinburgh press by using synchrotron X-ray radiography. Noteworthy, we conducted melt structure measurements of APR16 synthetic basalt at nearly the same HP-T conditions, at which viscosity was measured, using in situ multi-angle energy dispersive X-ray diffraction. Finally, the obtained results were used to estimate the mobility and ascent rate of anhydrous APR16-like magmas that we propose being representative of the primitive magmas feeding up the actual Campi Flegrei Volcanic District, and to reconstruct the depth at which their source might be located.

## Results

### Viscosity measurements of APR16 alkali basalt at HP-T by falling sphere technique

**Figure 1:**
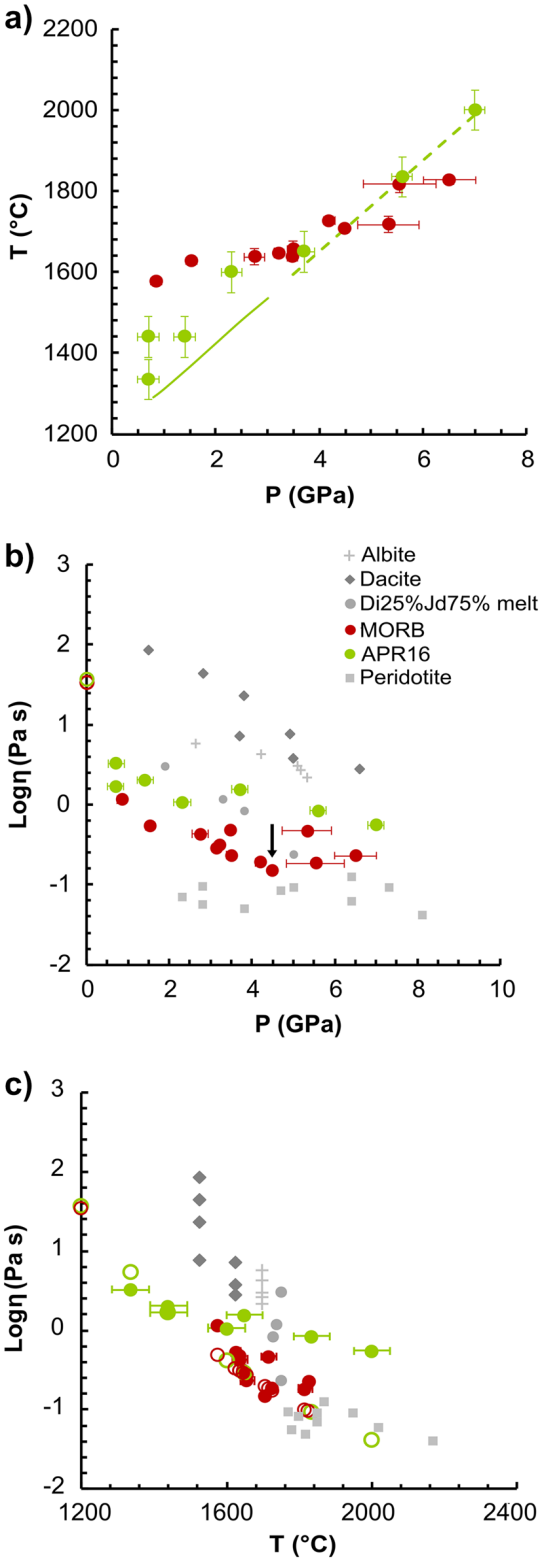
(**a**) Experimental P and T for APR16 (this study) and MORB^8^ experiments. The green line indicates the liquidus T obtained experimentally^19^ for APR16 using MELTS software^83^ and extrapolated to higher P (dotted segment). (**b**) and (**c**) Diagrams showing the logarithm of the measured viscosity on APR16 (this study) plotted as a function of (**b**) pressure (P) and (**c**) temperature (T) and compared with the experimental data available for peridotite^7^, MOR basalt^8^, diopside-jadeite (trachy-andesite^9^), albite^10^, and dacite^11^ compositions. The black arrow indicates the pressure at which a viscosity turnover was observed by Sakamaki et al.^8^ for MORBs and explained with the rapid densification (i.e. pressure-induced structural change). The empty circles (**b**,**c**) are the viscosities calculated for MORB and APR16 by using the model of Giordano et al*.*^5^ at ambient P and T of 1200 °C (**b**) and (**c**) at the same T of the experiments. Error bars for the viscosity data from this study are within the symbols size. The pressure and temperature uncertainties for our data are ± 0.2 GPa and ~ 50 °C^71^, respectively.

A total of seven successful viscosity measurements were performed respectively at 0.7 GPa/1335 °C, 0.7 GPa/1440 °C, 1.4 GPa/1440 °C, 2.3 GPa/1600 °C, 3.7 GPa/1650 °C, 5.6 GPa/1835 °C and 7.0 GPa/2000 °C (Fig. 1a and Table 1). Figure 1b,c summarize our determined viscosities plotted as function of pressure and temperature, which are compared with the experimental data available in literature for MOR basalts^8^, dacite^11^, albite^10^, diopside-jadeite (trachy-andesite^9^) and peridotite^7^ compositions. The viscosity for APR16 decreases from 3.266 (± 0.185) Pa s at 0.7 GPa/1335 °C and 0.552 (± 0.024) Pa s at 7.0 GPa/2000 °C (Table 1). A small arrow in Fig. 1b indicates a change in viscosity observed in the case of synthetic MOR basalts but not for APR16. Two isobaric runs at 0.7 GPa showed viscosities varying from 1.690 (± 0.126) to 3.266 (± 0.185) Pa s within a ΔT of 105 °C suggesting the T effect on the obtained viscosity values.

**Table 1:** Experimental conditions and result of viscosity measurements in this study.

Run	P (GPa)	T (°C)	Sphere diameter (mm)	Frame per second	Terminal velocity (mm/s)	Viscosity (Pa s)
APR16-PE3_top	0.7	1440	0.1130	23	0.0507	1.690 (± 0.126)
APR16-PE3_bottom	0.7	1335	0.1130	21	0.0507	3.266 (± 0.185)
APR16-PE2	1.4	1440	0.1700	21	0.0825	2.032 (± 0.092)
APR16-PE1	2.3	1600	0.1130	21	0.0848	1.064 (± 0.101)
APR16-PE7	3.7	1650	0.0867	21	0.0363	1.553 (± 0.156)
APR16-PE6	5.6	1835	0.1377	50	0.1500	0.840 (± 0.043)
APR16-PE5	7.0	2000	0.1402	50	0.2345	0.552 (± 0.024)

### Melt structure measurements

**Figure 2:**
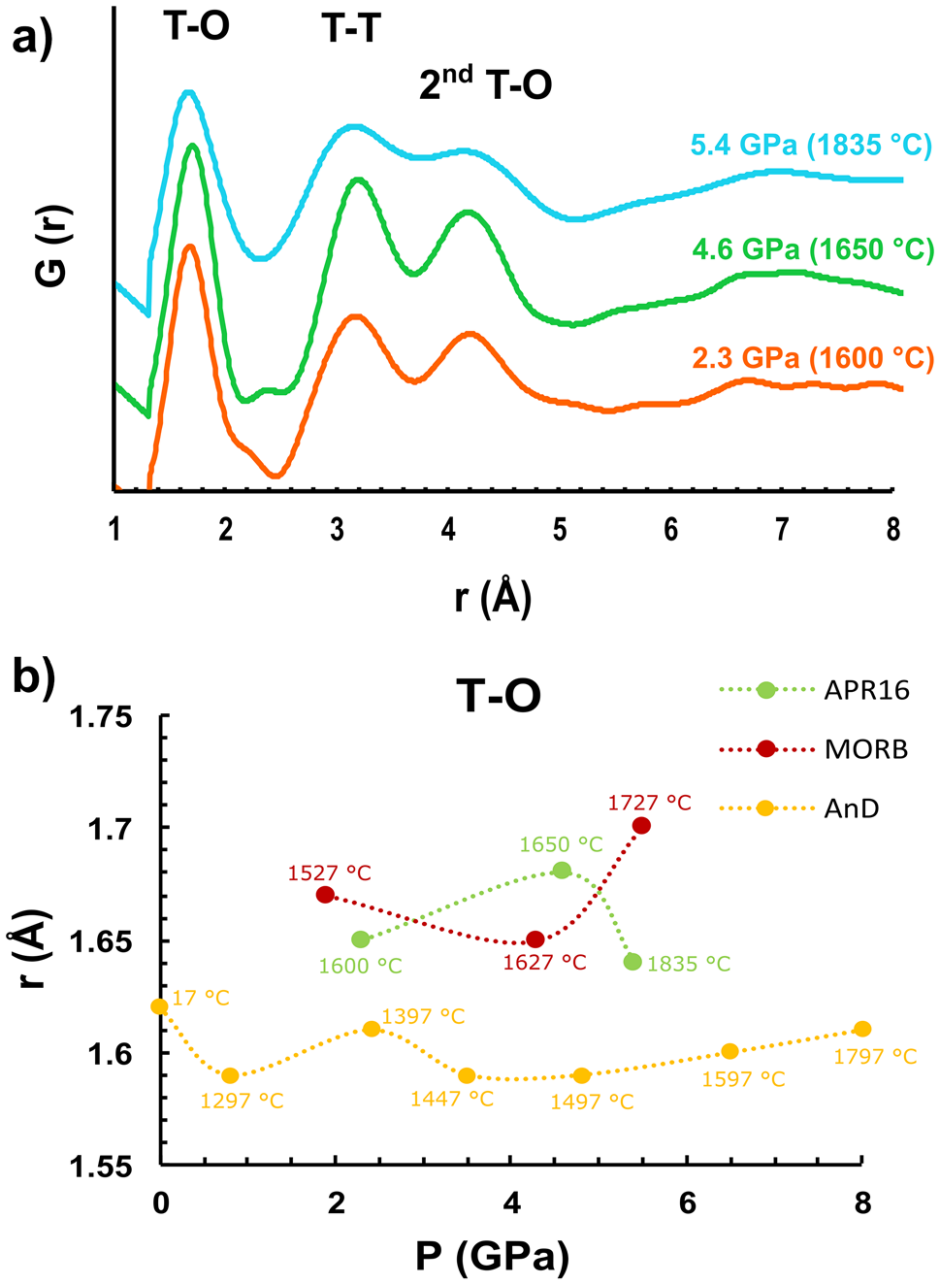
(**a**) Radial distribution function of the melt at the different investigated P of 2.3 GPa (1600 °C; orange), 4.6 GPa (1650 °C; green) and 5.4 GPa (1835 °C; light blue). The displayed peaks are relative to the tetrahedral-coordinated cation T-T, T-O and 2nd T-O interatomic distances. (**b**) T-O distance vs P for APR16 (in green) compared with data from Sakamaki et al*.*^8^ (in red) and de Grouchy et al.^25^ (in yellow).

The variation of viscosity of a magma during the ascent is likely to reflect changes in its interatomic distances as function of P and T. Hence, to better understand the rheological properties of APR16 in light of the atomic arrangement of the liquids at mantle conditions, melt structure measurements were performed immediately after the descent of the Pt sphere using the multi-angle energy-dispersive X-ray diffraction technique^23^ (see “Materials and methods” for details). To avoid crystallization caused by increasing thermal P of the cell during the three hours of acquisition, these measurements were performed at T about 20–30 °C higher than those at which the sphere was observed to fall, hence, above the liquidus conditions. This ensured X-ray diffraction patterns to be collected at conditions where the samples appeared totally molten over the duration of the measurements with minor change in the target P (Table 2) as determined by the equation of state of MgO surrounding the capsule. The structure factor, *S(Q)*, was obtained from the measured X-ray diffraction data using the in-house *aEDXD* analytical program (details as in “Materials and methods”) and converted to the pair distribution function *G(r)*. The melt structure parameters are listed in Table 2. Figure 2a shows the acquired spectra at 2.3 GPa/1600 °C, 4.6 GPa/1650 °C and 5.4 GPa/1835 °C. The displayed peaks are relative to the tetrahedrally coordinated cation-cation and cation–anion T-T and T-O interatomic distances. More in detail, according to previous studies^8,24^ the peaks located at 1.65 Å (2.3 GPa/1600 °C), 1.68 Å (4.6 GPa/1650 °C) and 1.64 Å (5.4 GPa/1835 °C) are assigned to the first peak of T-O bond length, the basic unit of the silicate melts. Figure 2b shows a comparison of the T-O distance plotted as function of P from this study along with that determined both by Sakamaki et al.^8^ in case of simplified MOR basalts and de Grouchy et al.^25^ for a melt with anorthite-diopside composition (Table 2). As it can be seen, APR16 basalt and synthetic MOR basalt have T-O distances that fall within a similar range at the experimental P–T conditions in contrast with the lower values reported by de Grouchy et al.^25^. Here, we observe an initial increase of T-O with P–T followed by a decrease that is in contrast with de Grouchy et al.^25^ and Sakamaki et al*.*^8^ showing an opposite trend between 2 and 5 GPa. The peaks at 3.13 Å (2.3 GPa/1600 °C), 3.16 Å (4.6 GPa/1650 °C) and 3.12 Å (5.4 GPa/1835 °C) correspond, instead, to the T-T bond length and reflect the distance between TO_4_ tetrahedron units. These peaks appear more clearly visible than in Sakamaki et al*.*^8^ and can be used to calculate T-O-T angle. Peaks at 4.10–4.15 Å correspond to the 2nd order T-O interatomic distance, according to Mysen^26^. With respect to the data by Sakamaki et al.^8^, although we observe consistent T-O and T-T distances, their trend is opposite, the latter for P < 4 GPa (Fig. S2a,b of Supplementary Material 1). We calculated the T-O-T angle (Table 2 and Fig. S2c of the Supplementary Material 1) using the relationship between T–O bond length and T–T bond length (T-O-T angle = 2arcsin{[T-T]/2[T-O]}) proposed in case of corner-sharing TO_4_ tetrahedra forming a 3D framework structure. We obtained the T-O-T angle of 143º at 2.3 GPa/1600 °C, 140º at 4.6 GPa/1650 °C, and 144º at 5.4 GPa/1835 °C. These data fall in the range of proposed averaged T-O-T angle of ~ 150° (Ref.^27^). Due to technical reasons, no data are available that show changes in the melt structure at isobaric or isothermal conditions. Therefore, any little variation can be interpreted as the result of the combined P–T effect on the melt structure that can affect the viscosity (Fig. 1b,c). In addition, possible effect on the melt structure owing to the different chemical composition is presented in the “Discussion” session.

**Table 2:** Experimental conditions of melt structure measurements in this and previous studies.

Run	P (GPa)	T (°C)	T-O length (Å)	T-T length (Å)	2nd T-O length (Å)	T-O-T (°)
**This study**						
APR16-PE1	2.3	1600	1.65	3.13	4.15	143
APR16-PE7	4.6	1650	1.68	3.16	4.14	140
APR16-PE6	5.4	1835	1.64	3.12	4.10	144
**Sakamaki et al.**^8^						
MOR basalt^(+)^	1.9	1527	1.67	3.20	4.20	147
MOR basalt^(+)^	4.3	1627	1.65	3.15	4.24	145
MOR basalt^(+)^	5.5	1727	1.70	3.10	4.12	132
**de Grouchy et al.**^25^						
AnD_Amb1	0.0	17	1.62	–	–	–
AnD_A1b	0.8	1297	1.59	–	–	–
AnD_A2b	2.4	1397	1.61	–	–	–
AnD_A3b	3.5	1447	1.59	–	–	–
AnD_A4b	4.8	1497	1.59	–	–	–
AnD_A5b	6.5	1597	1.60	–	–	–
AnD_A6b	8.0	1797	1.61	–	–	–

## Discussion

We calculated the viscosity for the simplified synthetic MOR basalt and APR16 liquid composition by using the model of Giordano et al*.*^5^ (GRD model) at ambient P and 1200 °C (H_2_O set to the minimum value of 0.02 wt% allowed by the model constraints). This resulted to be 33.7 and 37.2 Pa s, respectively (Fig. 1b) that is an order of magnitude higher than the viscosity determined experimentally at high P–T. Despite their similar 1-atm viscosity (i.e., the same viscosity determined for dacitic melts at upper mantle depths), this prediction highlights the important effect that P has on the viscosity of basaltic compositions. Since the T conditions of this study (1335–2000 °C) are controlled by the gradual increase of the liquidus T with increasing P (Fig. 1a) differently than Sakamaki et al*.*^8^, a better comparison of the viscosities between the two studies at higher P would be only possible by removing the effect of T. At this purpose, we extended the calculation using the GRD model of the 1-atm viscosity to the same experimental T conditions explored by Sakamaki et al.^8^ and this study (Fig. 1c) using the compositions of MORB^8^ and APR16 (Table S1 of the Supplementary Material 2). According to the VFT equation^28^ *log η (Pa s)* = *A* + *B(J mol*^*−1*^*)/[T(K) −* *C]*, the model provides A (pre-exponential factor representing the high-*T* limits to the viscosity), B and C coefficients of − 4.55, 5266.6 and 612.8 for APR16; while for MORB A = − 4.55, B = 5288.3 and C = 603.1. From Fig. 1c, the P effect on the viscosity can be inferred from the gradual increase of the difference between the calculated 1-atm viscosities (open symbols) and those determined experimentally from APR16 and MORB at high P (solid symbols). This difference becomes more and more evident in APR16 for P > 2 GPa, while it is negligible at lower P (see Fig. S3 of the Supplementary Material 1). Dramatic viscosity changes are, therefore, expected during the ascent of APR16-like melts even greater than MORBs as result of the P effect (Fig. 1). The increasing viscosity upon decompression will be further enhanced by shallow processes occurring within an open system (i.e., magma chambers and conduits) such as volatile exsolution and fractional crystallization.

The relation of viscosity with P is dissimilar between polymerized (NBO/T < 1) and depolymerized (NBO/T ≥ 2) melts as already noted by Wang et al*.*^29^. The viscosity of a diopsidic depolymerized melt^30^ increases with P, while that of polymerized melts such as MOR basalts^8^ (NBO/T = 0.7) first decreases, then, increases linearly with increasing P marked by a sudden change in the slope between 4 and 5 GPa (Figure 1 in Wang et al.^29^).

**Figure 3:**
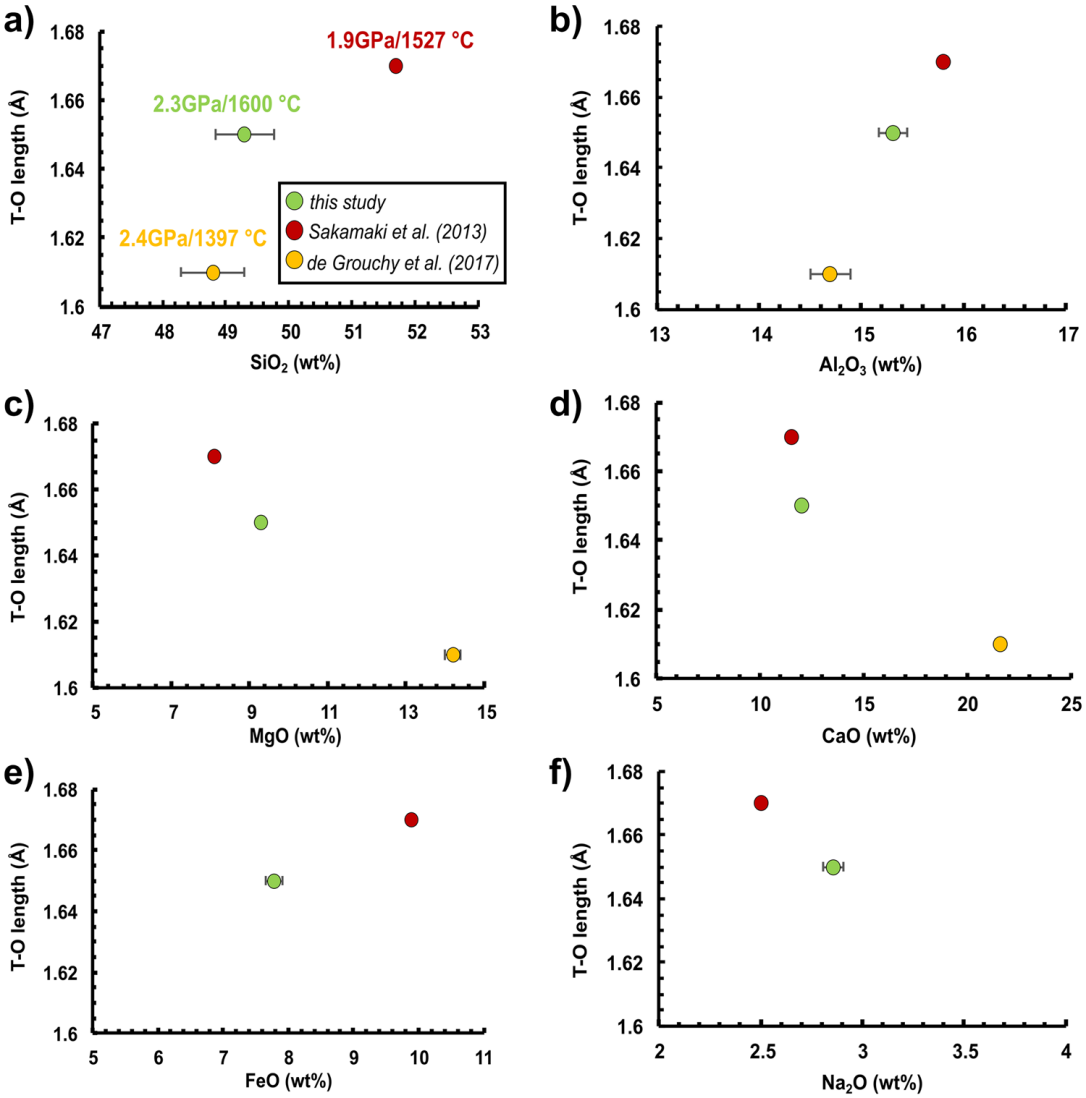
(**a**–**f**) Shown is the effect of major oxides on the T-O distance for APR16 compared with data from Sakamaki et al*.*^8^ (MORB) and de Grouchy et al.^25^ (AnD; anorthite-diopside composition) at ~ 2 GPa. Symbols are as in Fig. 2.

**Figure 4:**
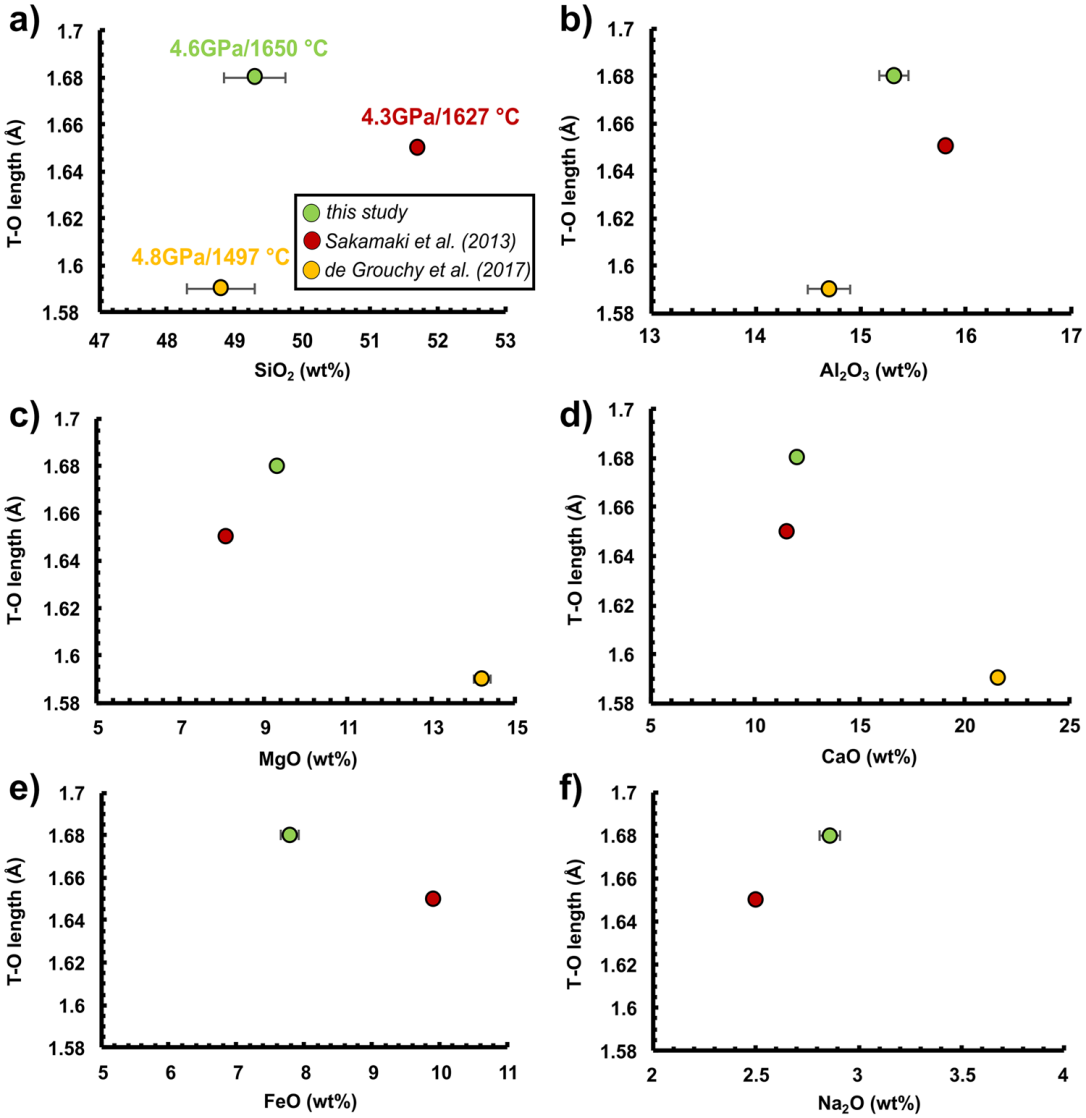
(**a**–**f**) Shown is the effect of each oxide component on the T-O distance for APR16 compared with data from Sakamaki et al*.*^8^ (MORB) and de Grouchy et al.^25^ (anorthite-diopside composition) at ~ 4.5 GPa. Symbols are as in Fig. 2.

The measured melt structure reflects the combined effect of P and T at each run as shown in Fig. 2b with the former being quite relevant (Fig. S3 of the Supplementary Material 1). In this regard, the availability of data on diverse basaltic compositions^8,25^ allows us also to explore the role that major oxides have on T–O bond length as reported in Figs. 3 and 4, respectively. As shown in Fig. 2, we observed different T–O bond length values for APR16 and MORB that are reversed at ~ 2 and ~ 4.5 GPa. This similar trend is reflected on the cation distances plotted versus the concentration of major oxides. Figures 3 and 4 show the effect of SiO_2_, Al_2_O_3_, MgO, CaO, FeO and Na_2_O on the T-O distance taken from the chemical compositions of the synthetic samples used in this study along with Sakamaki et al.^8^ and de Grouchy et al.^25^ at P of ~ 2 GPa (Fig. 3) and ~ 4.5 GPa (Fig. 4). The positive correlation between T-O and SiO_2_, Al_2_O_3_ and FeO content at ~ 2 GPa suggests their gradual incorporation into the tetrahedra with increasing oxide content. At ~ 4.5 GPa, the correlation is missing and, since T-O length does not correlate with Al_2_O_3_ content at ~ 4.5 GPa (Fig. 4b), it is difficult to attribute the difference in T-O length to Al coordination number change, although this cannot be neglected^8,27^. This opposite behavior also reflects to the T-O-T angle for P >  ~ 4.5 GPa (Fig. S2c of the Supplementary Material 1). The dependence of the viscosity from the structural parameters of the melt is reported in Fig. S4 of the Supplementary Material 1. Here the viscosity is calculated using the Eq. (1) (see below) at the P–T conditions at which the melt structure was measured. The calculated viscosity increases linearly with the T-T and T-O distances but decreases with the increase in T-O-T angle.

The results of this study are important to estimate the ascent rate of alkali basalts from the mantle source rock underneath Campi Flegrei and to shed light on the implications for the deep magma storage and supply. Generally, the volcanic hazard is linked to the explosivity of a volcano resulting from differentiation processes at shallow conditions and/or interaction with groundwater. However, there is evidence that the (re-)activation of volcanic areas is triggered by input of mantle-derived magmas resulting to rejuvenation of the plumbing system^31^ as observed for example in case of Etna^32,33^, Stromboli^34–36^, Kilauea^37,38^, Erebus^39^, Irazu^40^ and Turrialba volcanos^41^. This, in turn, implies that the eruptive style at surface is influenced by the physical and chemical conditions of magmas at depths of the upper mantle; accordingly, the knowledge of the viscosity and melt structure of magmas at conditions of the deep Earth can be a very useful information to improve the volcano monitoring activity. In the case of Campi Flegrei, several studies have pointed out the geochemical^42–44^ and seismic^45,46^ signature for the presence of mantle-derived magmas, the rheological properties of which remained unexplored so far. Indeed, only few experimental studies are available in literature on the rheological properties of magmas of interest for the Campi Flegrei Volcanic District (CFVD) but limited only to differentiated compositions from latites to trachytes at low (< 0.5 GPa) and atmospheric pressures^47–52^. CFVD, characterized by high density of population (about 550,000 inhabitants), is of particular interest among the most dangerous and, therefore, monitored volcanic areas with its multiple unrest episodes in the last 100 years^53^ accompanied by historical catastrophic eruptions^54^. Geochemical and geophysical observations like long-term record of degassing fumaroles, perturbation of the hydrothermal circulation^55^, chemistry and high temperatures of groundwaters^56^, and uplift/subsidence cycles^57^ have been interpreted as due to an evolving magmatic body extending down to sub-crustal depths^58^ that resulted in the last eruption occurred in 1538 (Mt. Nuovo^59^).

As mentioned above, our data can be used to model the mobility and ascent rate of alkaline basaltic melts (APR16) at upper mantle conditions and be integrated with further models^58,60–62^ (e.g., seismic, tomographic, geochemical) that have been used to reconstruct the plumbing system in the frame of volcanic hazard assessment. Recent petrographic and geochemical observations of the Campanian Ignimbrite products of Campi Flegrei supported by thermo-mechanical models of magma ascent provide evidence of magma chambers located at depths of ~ 7 km. The location of shallow magmatic chambers that cause unrest of the area has been matter of recent investigations for seismologists^45,46^ and geochemists^42,44,63^ that recognized the role of a deep magmatic input. On the other hand, there are geochemical evidences in favor of the presence of deep reservoirs at Moho level^20^. For these reasons, using our viscosity measurements, we calculated the melt parameters in the case of the primitive APR16 magma^13,64,65^ (a valid candidate of deeply stored magma underneath Campi Flegrei), such as mobility (defined as *Δρ/η,* where *Δρ* is the density contrast between the melt and the surrounding permeable crystalline matrix, and *η* the melt viscosity) and ascent rate *w*_*0*_ from the source rock (~ 60–80 km of depth^66^) up to the magma reservoir located at the Moho level (~ 25 km of depth in the Campi Flegrei area^14,58,61,64,67–69^). At this aim, first we fitted our data to an Arrhenian equation,

1 $$log\eta =A+\left(\frac{B+{B}_{p} \cdot (P-1)}{T}\right)$$

where *η* is the viscosity in Pa s, *T* the absolute temperature in Kelvin, *A* is assumed to be a constant (− 4.5), *P* is the pressure in bars, and *B* and *B*_*p*_ are fit coefficients (Ref.^70^). The values of *B* = 7909.26 and *B*_*p*_ = 0.02575 easily reproduce the data to within experimental error (see Fig. S5 of the Supplementary Material 1). The positive *B*_*p*_ term indicates an increase in viscosity with increasing P (Ref.^70^) and confirm our previous considerations (see Fig. 1c).

We used the equation^71,72^,

2 $$\phi {w}_{0}=\frac{kg\Delta \rho }{\eta },$$

with,

3 $$k=\frac{{a}^{2}{\phi }^{n}}{C},$$

where *k* is the permeability, *a* is the characteristic crystal size (m), $\phi$ is the melt fraction (expressed with values from 0 to 1 according to McKenzie^73^), *n* and *C* are numerical constants^71^, *g* is the gravitational acceleration constant (9.81 m s^−2^), and ${w}_{0}$ is the melt ascent velocity (m yr^−1^). The permeability was calculated from parameters of mantle rocks as proposed in literature such as *a* = 0.002 m^71^, $\phi$ = 0.05, 0.1 and 0.15, *n* = 3 and C = 1000^74^. An approximation for the density of the APR16 melt is taken by Agee^15^ by analogy with the reported basaltic composition, while the density of the rock matrix is given by Fedi et al*.*^58^. We obtained a melt mobility of ~ 0.15–0.4 g cm^−3^ Pa^−1^ s^−1^. The calculated averaged ascent velocity of alkaline melts from the source to the Moho is about 0.1, 1.5 and 6.0 m yr^−1^ by assuming a wide range of melt fractions of 1, 5 and 10%, respectively. A more realistic value of melt fraction is provided by Mazzeo et al.^66^ who show that a fraction of 6–7% of partial melting of a phlogopite-bearing enriched source is required to generate a melt with trace elements and Sr–Nd-isotopic features matching those of high-Mg, K-basalts of Procida Island (the type locality of APR16). The calculated averaged ascent velocity of APR16-like alkaline melt from the source to the Moho is about 2.5 m yr^−1^. The presence of phlogopite in the source rocks implies that a small amount of water can be dissolved in the primitive APR16 melt (up to 2 wt%)^14^. To date, the effect of H_2_O on the viscosity of silicate melts at high P–T has not been well understood yet. It is, however, expected that small amount of water would cause the viscosity to further decrease at the mantle source with implication for the rheology of magmas. A preliminary consideration comes from the modelled viscosity of APR16 calculated at 1 atm and 1300 °C assuming different amount of H_2_O (e.g., 0.02, 0.5, 1, 1.5 and 2). The viscosity decreases within 1 log unit and, therefore, compensated by the predicted increase of viscosity with P at fixed T as discussed above.

**Figure 5:**
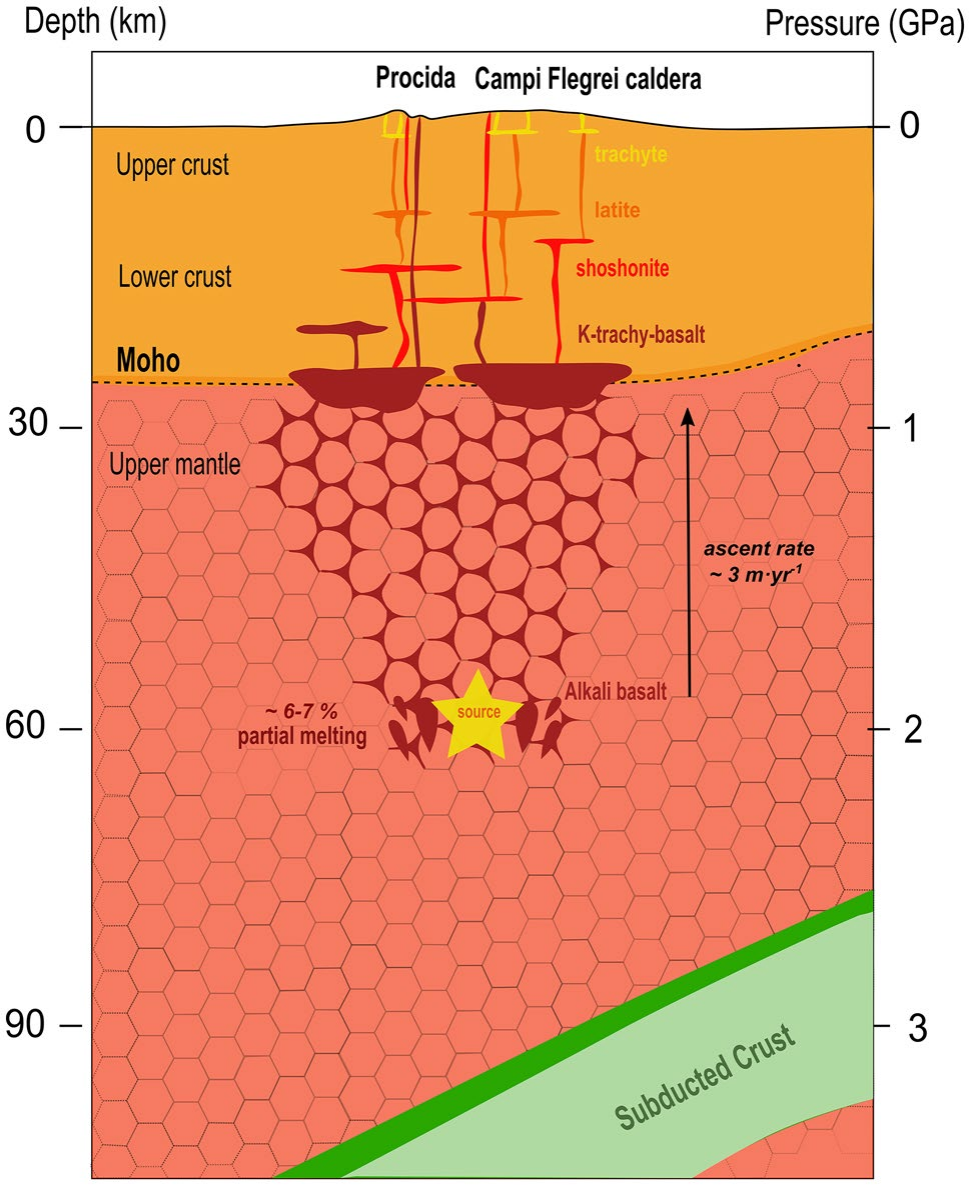
Cartoon summarizing the depth at which APR16 melt form^66^ (yellow star), its migration path (red stripes) and evolution in terms of chemistry and rheology within a subduction geodynamic setting. CFVD plumbing system from the Moho level towards the surface has been modified after Fedi et al*.*^58^.

A time interval (*Δt* ~ 20 ka) between the major historical eruptions occurred at Campi Flegrei (Green Tuff, 55 ka; Campanian Ignimbrite, 39 ka; Neapolitan Yellow Tuff, 15 ka), along with an average ascent velocity of ~ 2.5–3.0 m yr^−1^ (corresponding to a ~ 6–7% degree of partial melting of the source rock) would set the onset for magma mobilization upwards at a depth of ~ 60 km (± 20 km for an ascent velocity varying up to 1 m yr^−1^) (Fig. 5). This depth is consistent with what proposed on geochemical basis by Mazzeo et al.^66^ to explain the generation of Procida primitive basaltic magmas and in agreement with natural constrains such as the presence of a magma reservoir at about 25 km of depth^61,64,67–69^ and the imaged subducting slab located at depths of ~ 100–150 km^75^.

## Materials and methods

### Starting material

The choice of APR16 for this study is enhanced by the fact that this sample differs from the other silicate melts investigated in literature so far (e.g., basaltic^8^) for both the chemical composition (i.e., alkaline basalt) and the geodynamic setting in which it originated (i.e., subduction related). Moreover, this sample represents the near-primary melt of the CFVD^13,76^. The chemical composition is reported in Table S1 of the Supplementary Material 2 along with the chemical composition of the synthetic glass used for the viscosity and melt structure measurements. According to the Total Alkali-Silica (TAS) diagram (see Fig. S1 of the Supplementary Material 1), APR16 can be classified as a basalt high in alkali content (4.47 wt%). The synthetic glass was obtained by melting the initial natural APR16 powder at 1400 °C for 15 min at ambient pressure and an atmosphere buffered by a mixture of CO and CO_2_ fluxed inside the furnace to the nickel-nickel oxide *f*_O2_ level (refs^17–20^ for details). The starting material is a brownish crystal-free glass as confirmed by both microprobe and BSE images (Fig. S6 of the Supplementary Material 1). The Fe^3+^/∑Fe of the quenched glass is 0.24 (± 0.04; Fig. S7 of the Supplementary Material 1) determined using the Mössbauer spectroscopy available at the Department of Earth Science in Sapienza University (Rome). This value would set an intrinsic *f*o_2_ ranging between − 5.4 and − 0.6 log units for the melt calculated using the model by Sack et al*.*^77^ within the experimental conditions at least for the (fast) viscosity measurements.

### High-pressure and high-temperature experiments

Experiments were performed using the VX-3 Paris–Edinburgh (PE) press^78,79^ available at the 16BM-B beamline at the Advanced Photon Source (Argonne National Lab, Illinois^71^). The cell assembly designed for viscosity and melt structure measurements consisted of boron–epoxy (BE) gaskets, a MgO ring, ZrO_2_ caps, a graphite heater, a BN outer container and a graphite capsule^71^. The graphite capsule encloses a cylindrical sample 1.2 mm of diameter and 2 mm of height. The MgO ring is placed between BE gasket and graphite heater to increase stability of the cell assembly and maintain anvil gap. The MgO ring was used as the pressure marker through the use of acquired X-ray diffraction peaks and fitted with the proper equation of state^80^. The uncertainty on the calculated pressure is typically of ~ 0.15 GPa. The loaded cell was first compressed to the target pressure by a hydraulic system connected to the Paris-Edinburgh press. After compression, the sample was heated quickly at a rate of ~ 100 °C/s above the liquidus^14^ and the temperature estimated using a power vs. temperature calibration curve reported by Kono et al*.*^23^. The achievement of the liquidus conditions was confirmed both by the straight fall of the Pt sphere through the capsule and the disappearance of peaks in the XRD patterns. Finally, the experiments were quenched by shutting down the electrical power to the heater.

### Ultrafast X-ray imaging and the falling-sphere viscosity measurement

The melt viscosity (Pa s) was calculated from the measured fall velocity of the Pt sphere through the silicate liquid using the Stokes law that incorporates the proper correction factors for the wall (*F*, 3rd order Faxen correction factor^81^) and the end effect (*E*)^12^

4 $$\eta =\frac{g{d}_{s}^{2}\left({\rho }_{s}-{\rho }_{l}\right)}{18\upsilon }\frac{F}{E}$$

5 $$F=1-2.104\left(\frac{{d}_{s}}{{d}_{l}}\right)+2.09{\left(\frac{{d}_{s}}{{d}_{l}}\right)}^{3}-0.95{\left(\frac{{d}_{s}}{{d}_{l}}\right)}^{5}$$

6 $$E=1+\frac{9}{8}\frac{{d}_{s}}{2Z}+{\left(\frac{9}{8}\frac{{d}_{s}}{2Z}\right)}^{2} ,$$

where *ν* is the terminal velocity (mm s^−1^) of the probing Pt sphere, *ρ* is its density (g cm^−3^) and *d* the diameter (mm), with *s* and *l* subscripts denoting properties of the sphere and liquid, respectively. The liquid diameter, *d*_*l*_ and its height (*Z*) are determined from the capsule dimensions of 1.2 mm and 2 mm, respectively, that are also observed by radiography to not change during the experiments. The other parameters are *ρ*_*s*_ = 19.4 g cm^−3^; *ρ*_*l*_ = 2.8 g cm^−3^; *d*_*s*_ = 0.08–0.17 mm (Table 1). A fixed value of 2.8 g cm^−3^ was chosen for the density (*ρ*_*l*_) of molten APR16 according to the work by Sakamaki et al.^8^ considering that a difference up to ± 1 g cm^−3^ would affect the viscosity value by 0.1 Pa s and, however, a variation of less than 0.5 g cm^−3^ was reported within a similar P range^8^. The diameter of the spheres was measured by X-ray radiography using the high-resolution Prosilica GC1380 camera with a resolution of 0.85 µm per pixel^23^. The uncertainty in the diameter of the probing sphere contributes to errors in viscosity due to the small sphere size. The high-resolution X-ray radiography has a ± 2 µm resolution in imaging^23^, which causes ± 2.1–4.1% uncertainty in viscosity. Therefore, the overall uncertainty in the viscosity determination is <  ± 9.3%. Experimental measurements of the viscosity were performed using unfocused white X-ray beam with radiographic images captured by a high-speed camera (Photron FASTCAM SA3) with 250 and 500 frame per second recording time (250 fps for the runs APR16-PE5, PE6, PE7; 500 fps for the runs APR16-PE1, PE2 and PE3) with a resolution of 2.5 µm per pixel. Supplementary Movie 1 shows the falling Pt sphere in APR16 melt at ~ 1.4 GPa and ~ 1440 °C in real time. The radiographic images were collected during heating of the run until the fall of the sphere could be observed. The falling sphere velocity, necessary to determine the viscosity using Stokes equation, was determined from the collected radiographic images with the Tracker plugin in the ImageJ software package (Table 1 and Figs. S8–S14 of the Supplementary Materials).

### Melt structure measurements

Liquid structure studies are conducted by the multi-angle energy-dispersive X-ray diffraction technique. A large Huber stage holding a Ge solid state detector (Ge-SSD) allows precise control 2θ angle from 2° to 39.5°. For diffracted X-rays, two slits are located on the diffractometer arm: one near the sample (around 60 mm from the center) and the other in front of the detector (at around 480 mm distance). The detector arm also supports the Ge-SSD with a liquid nitrogen dewar. The collimation depth control is important to liquid scattering measurement for discriminating sample signal from background scattering caused by surrounding materials. Diffraction patterns were collected at 10 fixed diffraction angles (2θ = 3°, 4°, 5°, 7°, 9°, 12°, 16°, 22°, 28°, 35°) to cover a wide range of scattering vectors (Q). More details of the processing of the data are described in Yamada et al.^82^. The peaks position, finally, was obtained by Gaussian fitting of the peaks.

## Supplementary Information

Supplementary Figures.

Supplementary Table S1.

Supplementary Video S1.

## Data Availability

The data that support the findings of this study are available within the article, its Supplementary Information files and from the corresponding author upon request.
