# Supplementary material for: Experimental measurements of the viscosity and melt structure of alkali basalts at high pressure and temperature
Source: Sci Rep. 2022 Feb 16;12:2599. doi: 10.1038/s41598-022-06551-7 (PMC8850584; doi:10.1038/s41598-022-06551-7)
Supplement: Supplementary file 1 — Supplementary Figures. [file 41598_2022_6551_MOESM1_ESM.pdf]

Supplementary Figures for

Experimental measurements of the viscosity and melt structure of alkali basalts at high pressure and temperature

Barbara Bonechi[1*], Vincenzo Stagno[1,2], Yoshio Kono[3], Rostislav Hrubiak[4], Luca Ziberna[5,6], Giovanni B. Andreozzi[1], Cristina Perinelli[1], Mario Gaeta[1]

1 Dipartimento di Scienze della Terra, Sapienza Università di Roma, P.le Aldo Moro, 5 00185 Rome, Italy;
2 Istituto Nazionale di Geofisica e Vulcanologia, 00143 Rome, Italy;
3 Geodynamics Research Center, Ehime University, 790-8577 Matsuyama, Japan;
4 High Pressure Collaborative Access Team, X-ray Science Division, Argonne National Laboratory, 9700 S. Cass Avenue, Argonne, IL 60439, United States;
5 Dipartimento di Matematica e Geoscienze, Università degli Studi di Trieste, Via Weiss 8, 34128 Trieste, Italy.
6 Bayerisches Geoinstitut, University of Bayreuth, 95440 Bayreuth, Germany.

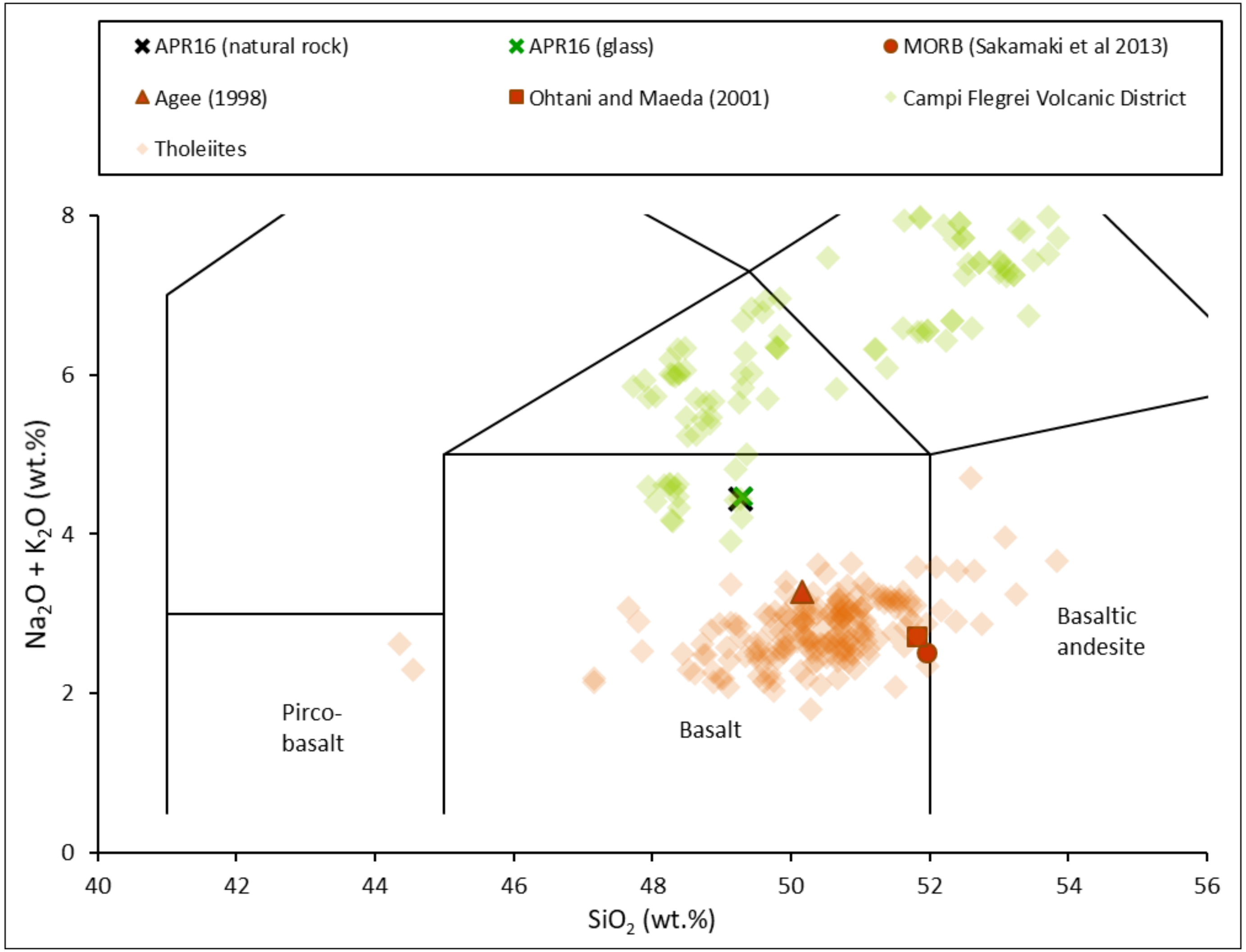

Figure S1 Total Alkali-Silica diagram showing the APR16 starting material compared with synthetic and natural basaltic compositions used in previous studies as reported in Table S1. Data for Campi Flegrei Volcanic District (green diamonds) and for Tholeiites (orange diamonds) are from GEOROC database (http://georoc.mpch-mainz.gwdg.de/georoc/Start.asp)

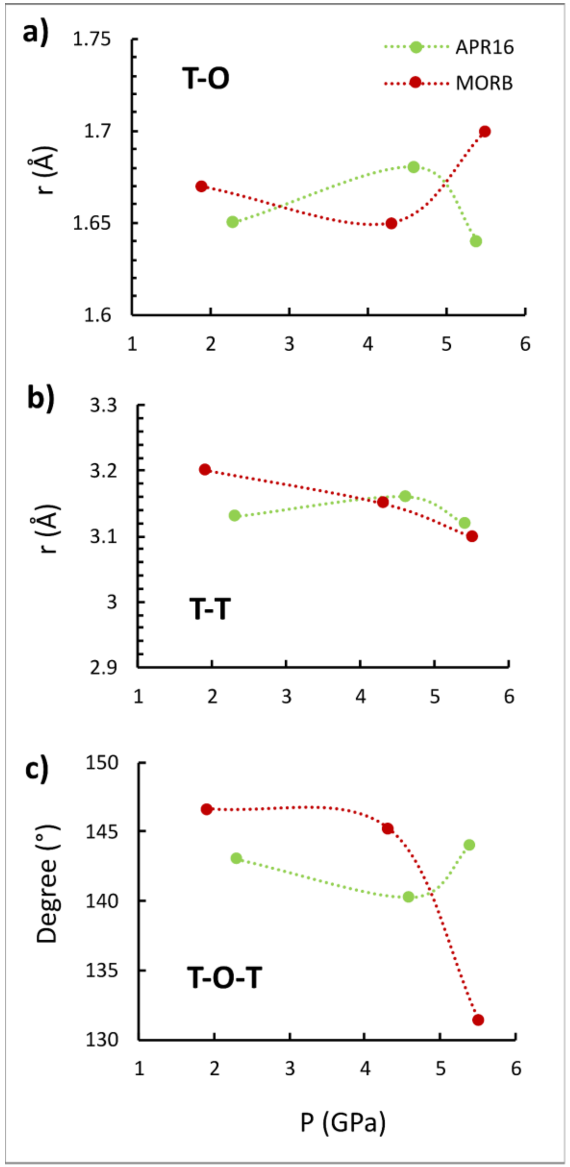

Figure S2 Atomic distance (a-b) and T-O-T angle (c) vs pressure.

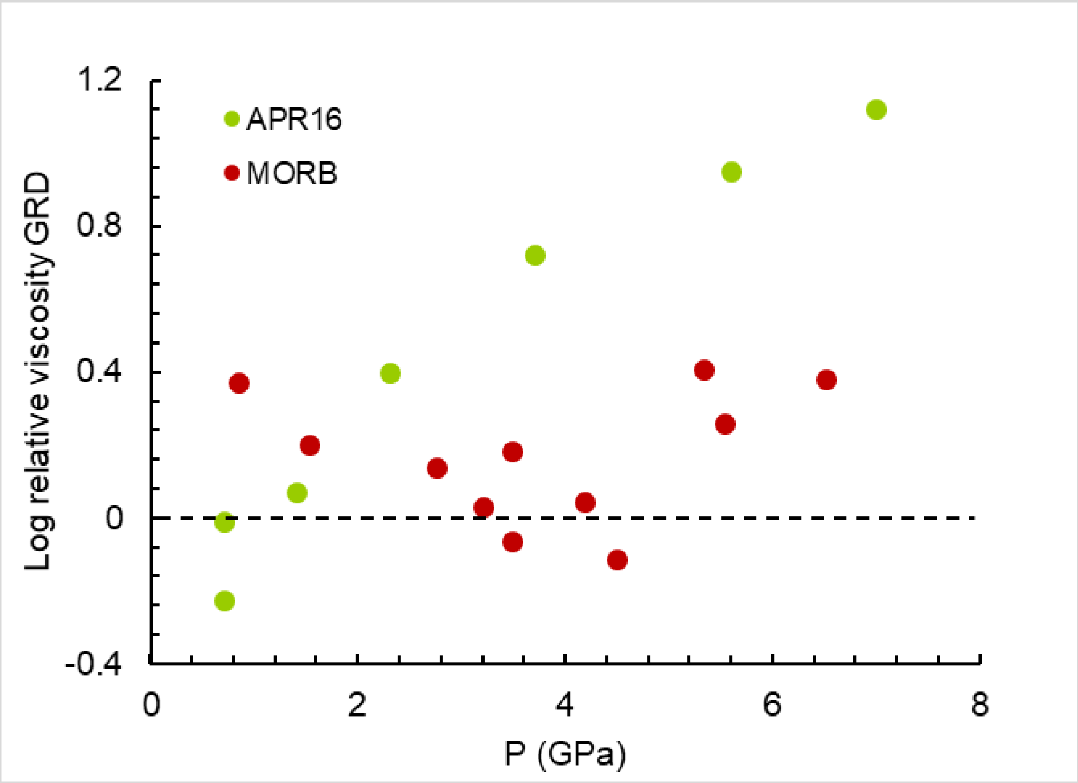

Figure S3 Logarithm of the relative viscosity (i.e., the difference between the viscosity measured for APR16 and MORB and their 1-atm viscosities calculated through the model by Giordano et al.[5] within the same T range).

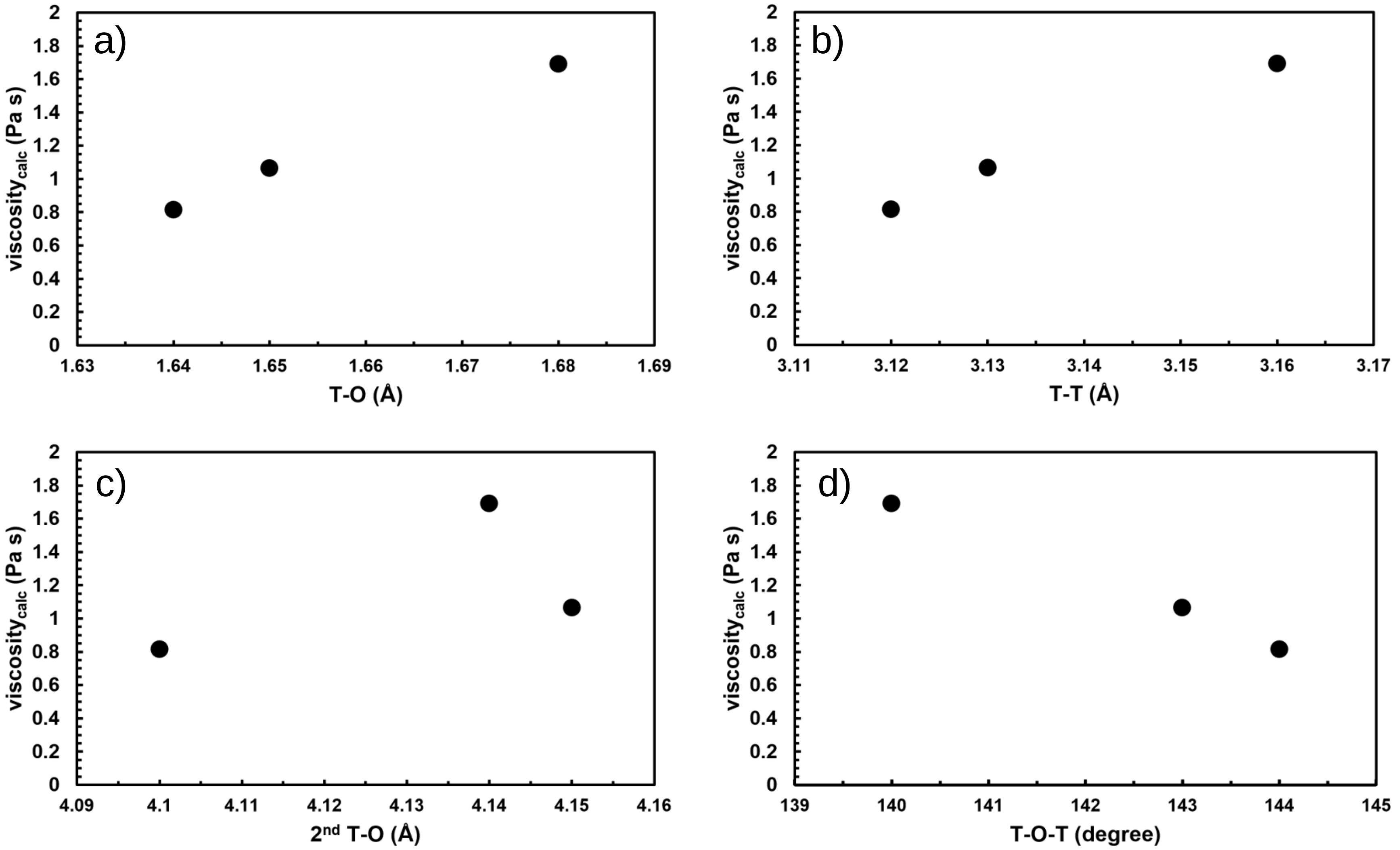

Figure S4 The viscosity of APR16 calculated using (eq1) plotted vs the atomic distances (a-c) and T-O-T angle (d). The viscosity is plotted at the P-T conditions at which the melt structure was measured (Table 2).

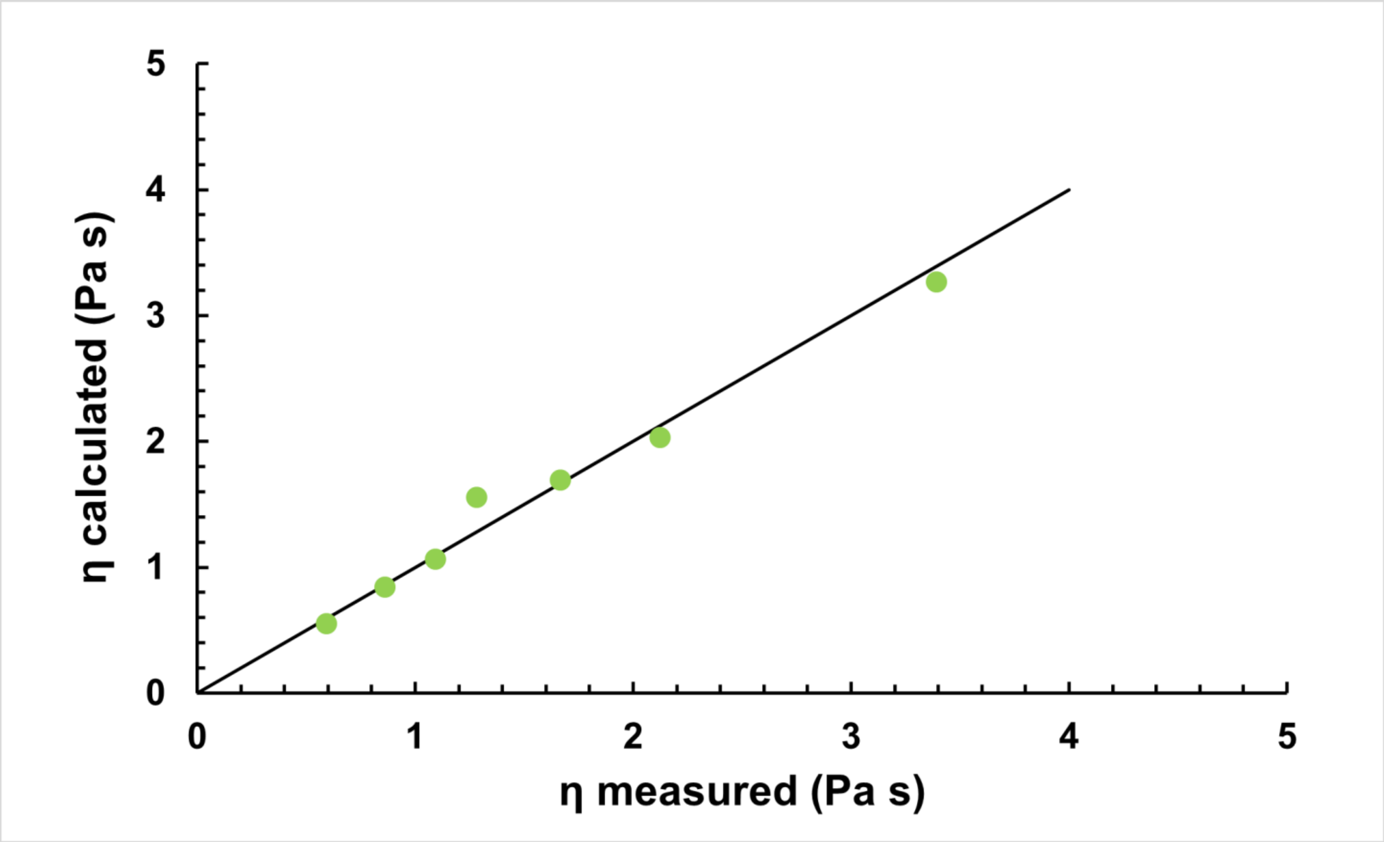

Figure S5. Comparison between the measured absolute viscosity for APR16 and the viscosity calculated using their Arrhenian fitting equation (Eq. 1 in the text).

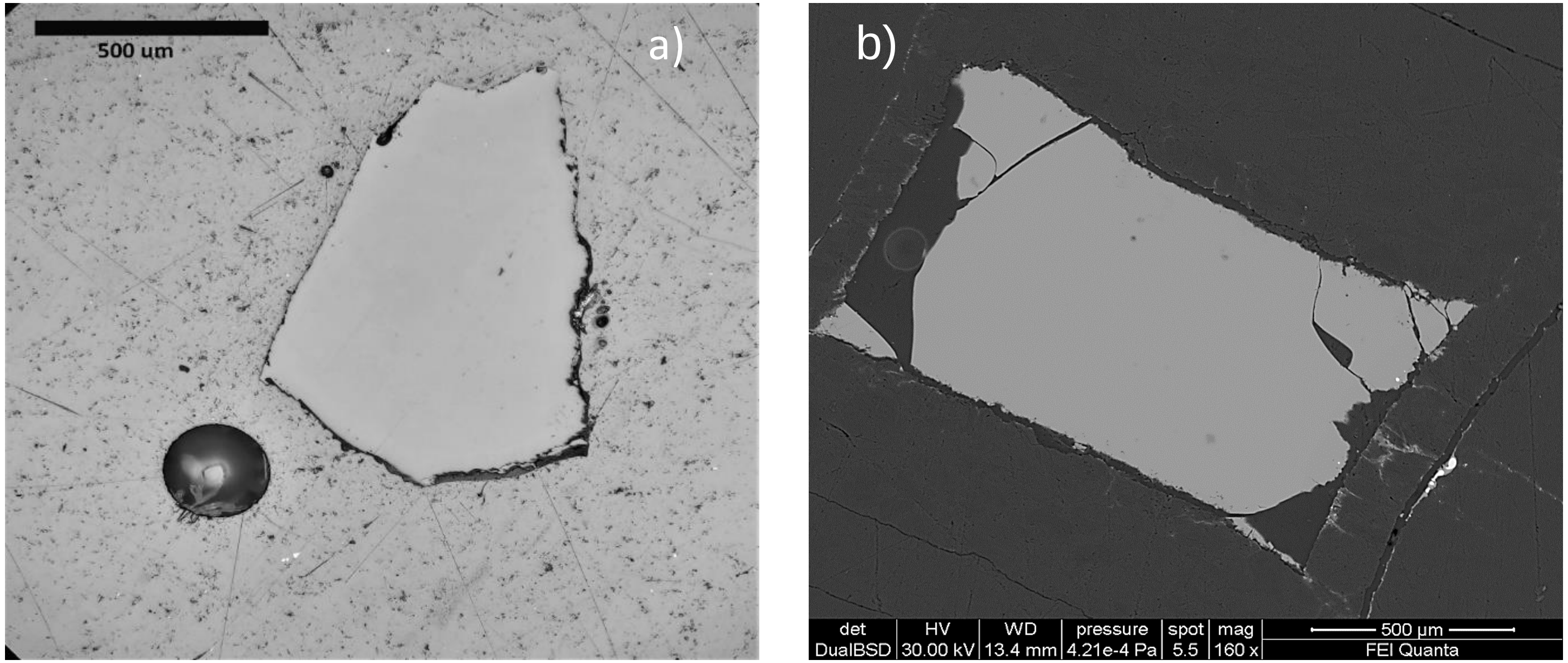

Figure S6 a) optical microscope image of APR16 starting material, and b) back-scattered electron image of APR16-PE6 run (1835 °C, 5.6 GPa) showing the chemical and textural homogeneity.

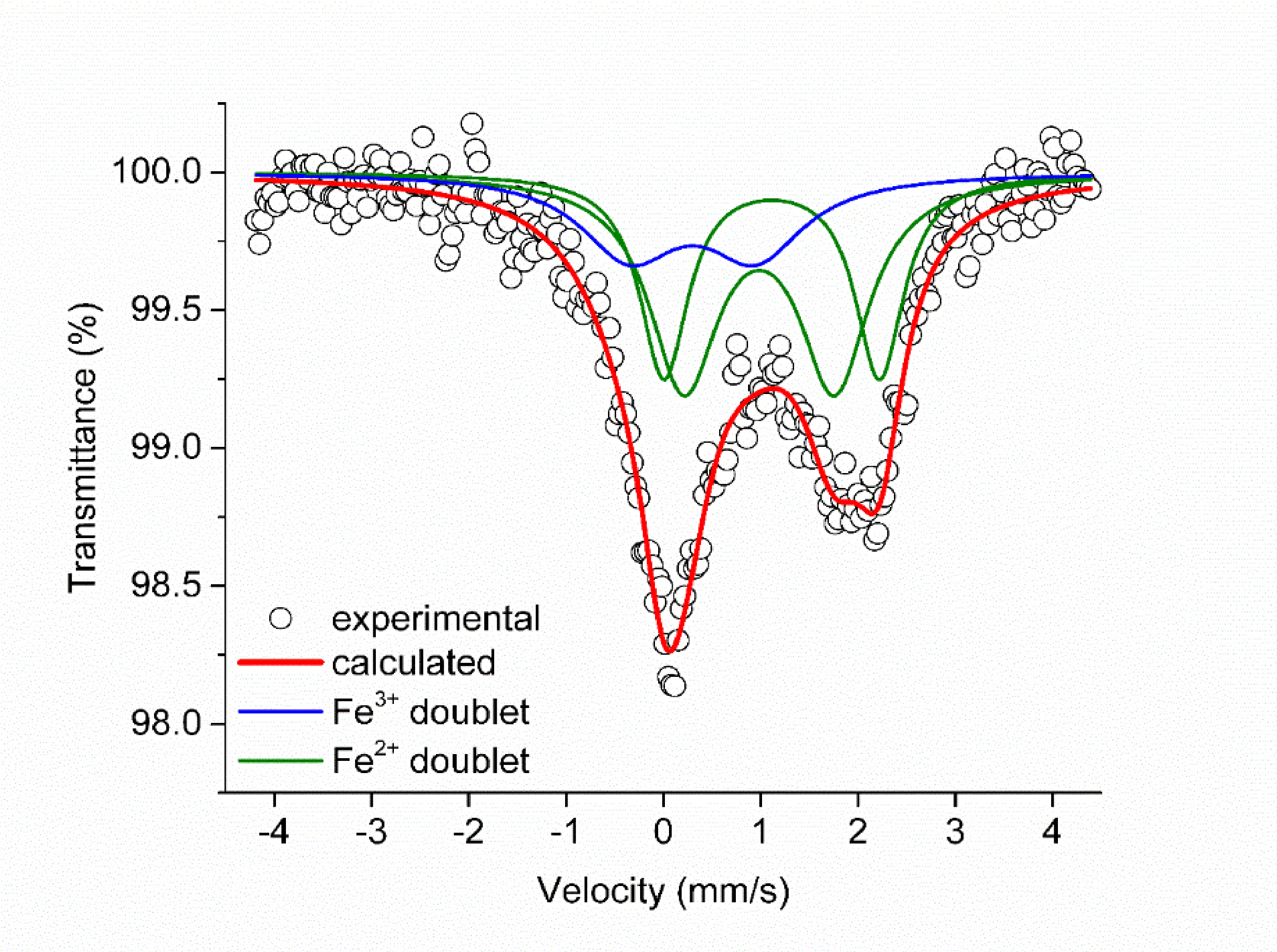

Figure S7 Mössbauer spectrum of APR16 starting glass.

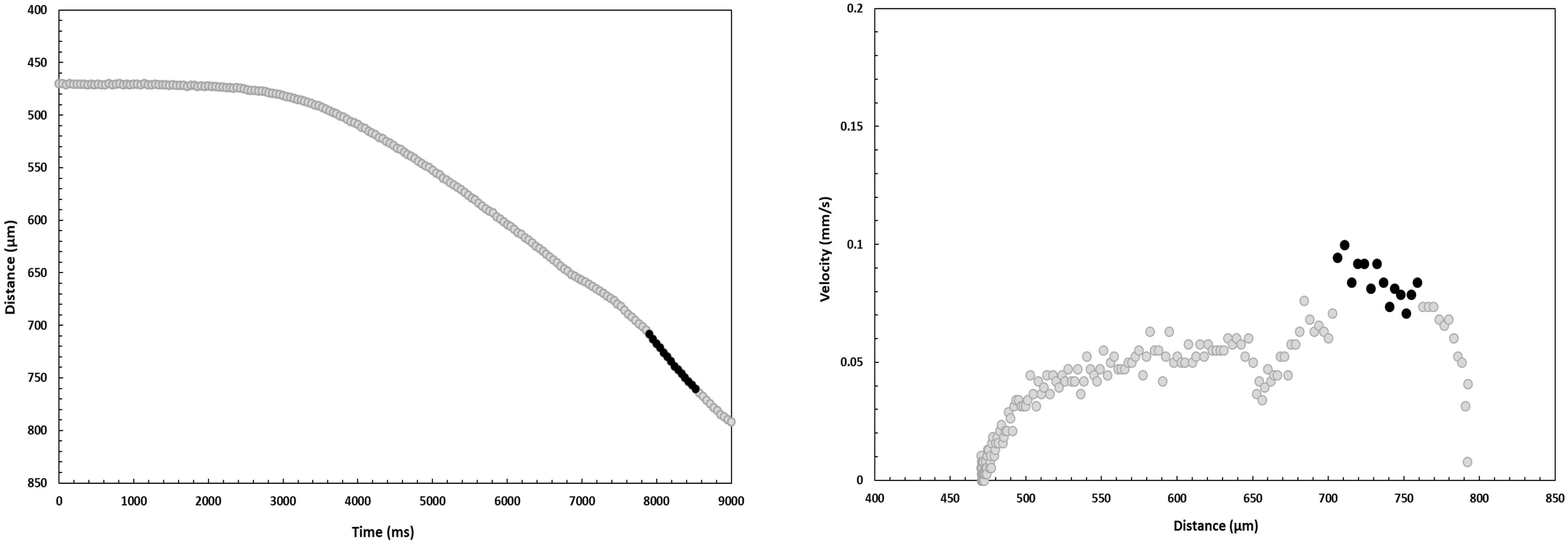

Figure S8 Diagrams of the falling distance versus time (left) and falling velocity versus distance (right) of the Pt sphere (diameter of 113 µm) for the run at 2.3 GPa and 1600 °C (APR16-PE1). The terminal velocity was calculated by averaging an interval of maximum velocities (black dots) achieved only at limited regions of falling distance (~700–770 µm distance).

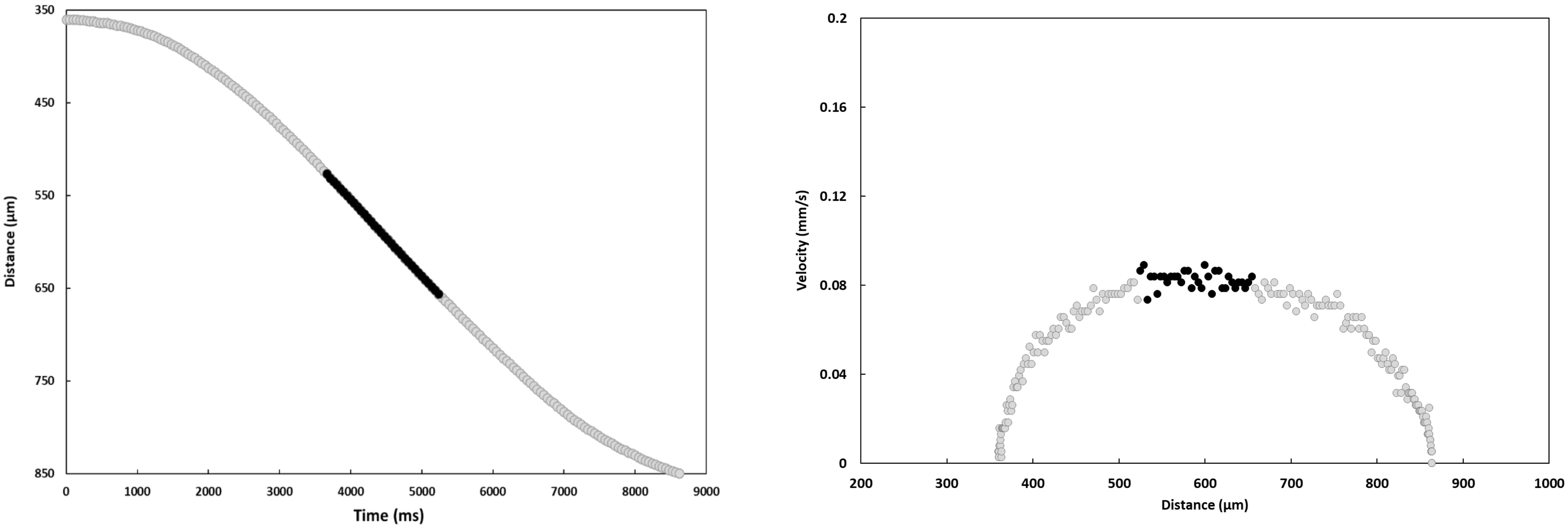

Figure S9 Plots of the falling distance as function of time (left) and falling velocity versus distance (right) of the Pt sphere (diameter of 170 μm) for the run at 1.4 GPa and 1440 °C (APR16-PE2). The terminal velocity was calculated by averaging an interval of maximum velocities (black dots) achieved only at limited regions of falling distance (~490–660 μm distance).

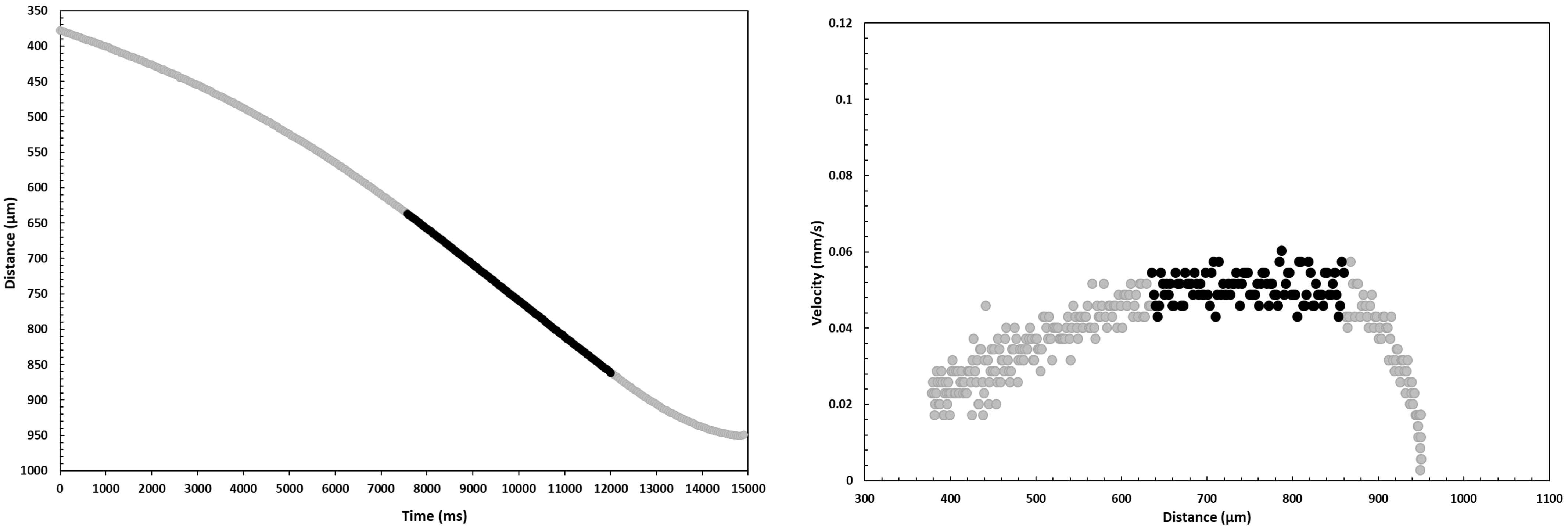

Figure S10 The falling distance as function of time (left) and velocity (right) of the Pt sphere (diameter of 107 µm) in run at 0.7 GPa and 1440 °C (APR16-PE3 top). The terminal velocity was calculated by averaging an interval of maximum velocities (black dots) achieved only at limited regions of falling distance (~650–850 µm distance).

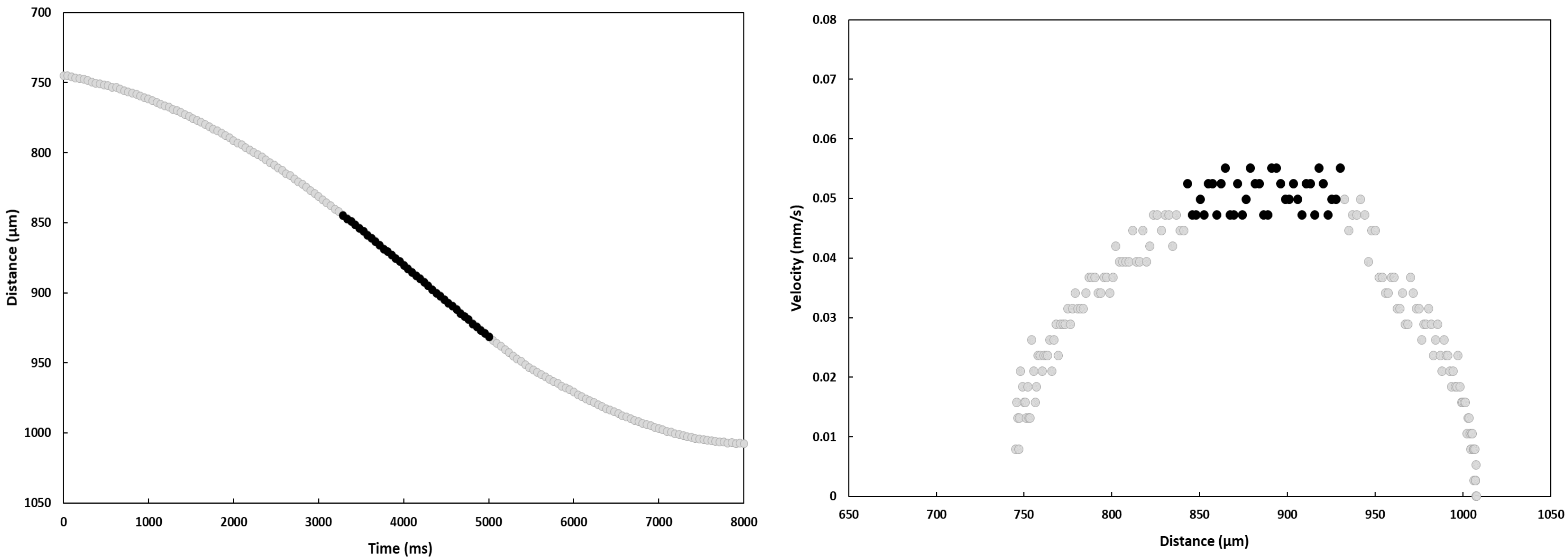

Figure S11 Falling distance versus time (left) and velocity (right) of the Pt sphere (diameter of 156 µm) recorded for the run at 0.7 GPa and 1335 °C (APR16-PE3 bottom). The terminal velocity was calculated by averaging an interval of maximum velocities (black dots) achieved only at limited regions of falling distance (~850–950 µm distance).

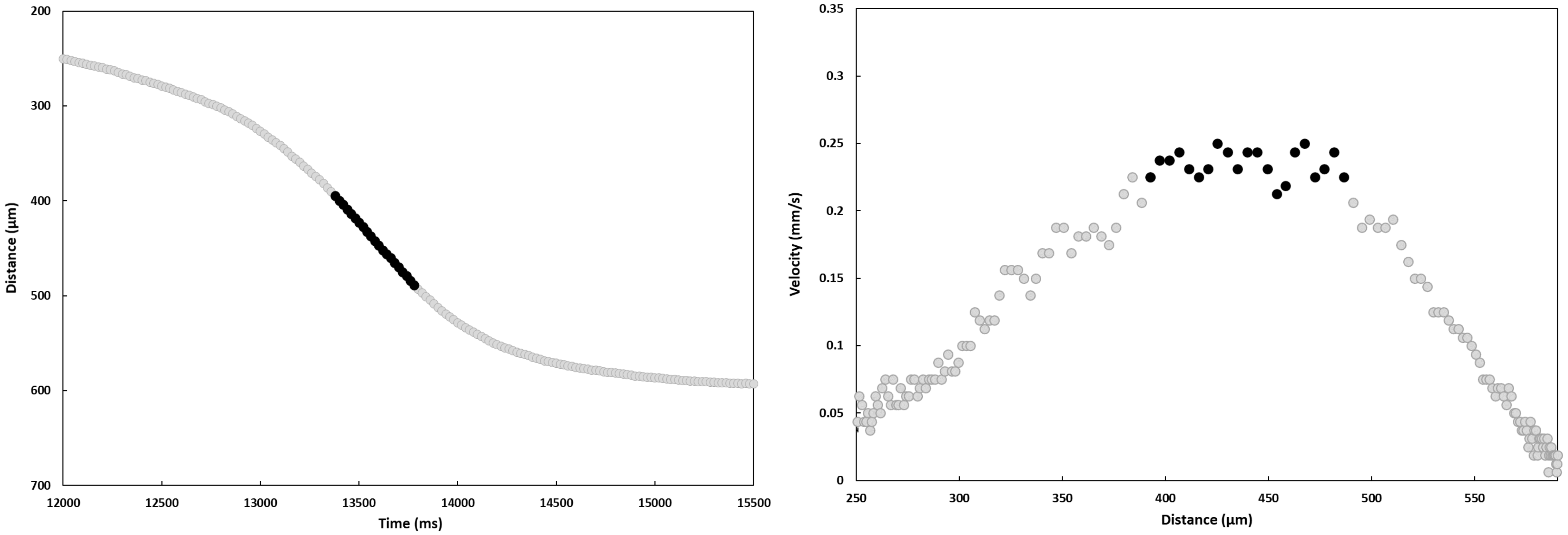

Figure S12 The falling vertical distance (left) and velocity profile (right) of the Pt sphere (diameter of 140 µm) for the run at 7 GPa and 2000 °C (APR16-PE5). The terminal velocity was calculated by averaging an interval of maximum velocities (black dots) achieved only at limited regions of falling distance (~400–500 µm distance).

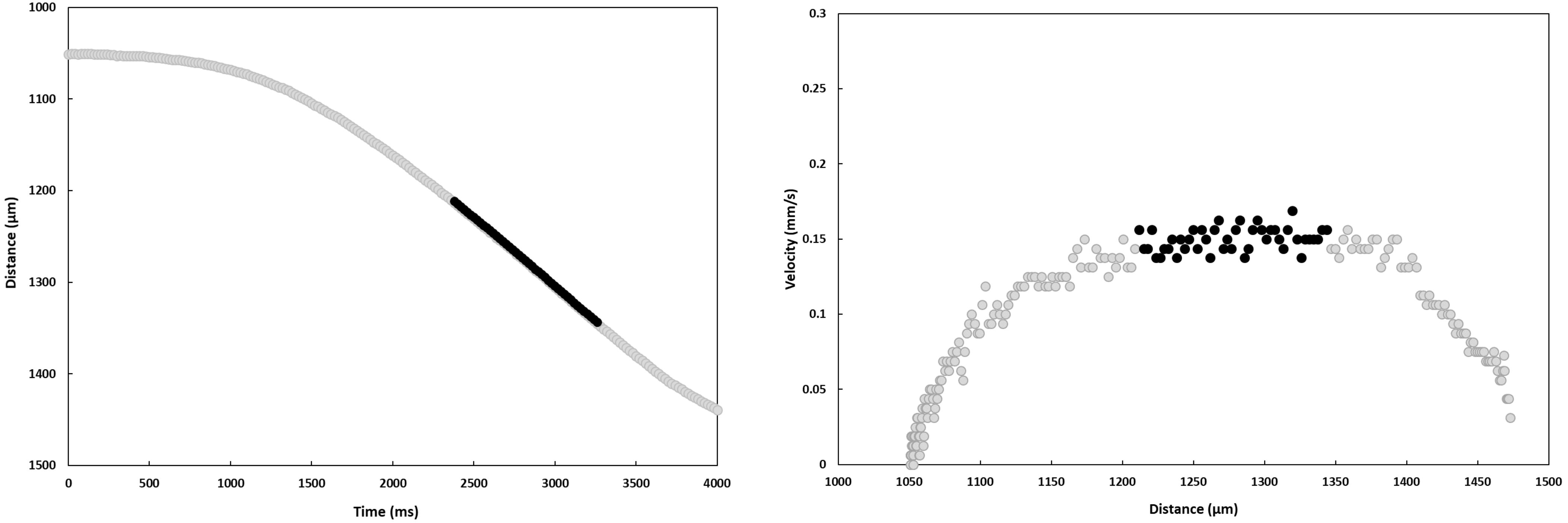

Figure S13 The falling distance versus time (left) and velocity profile (right) of the Pt sphere (diameter of 138 μm) for the run at 5.6 GPa and 1835 °C (APR16-PE6). The terminal velocity was calculated by averaging an interval of maximum velocities (black dots) achieved only at limited regions of falling distance (~1200–1350 μm distance).

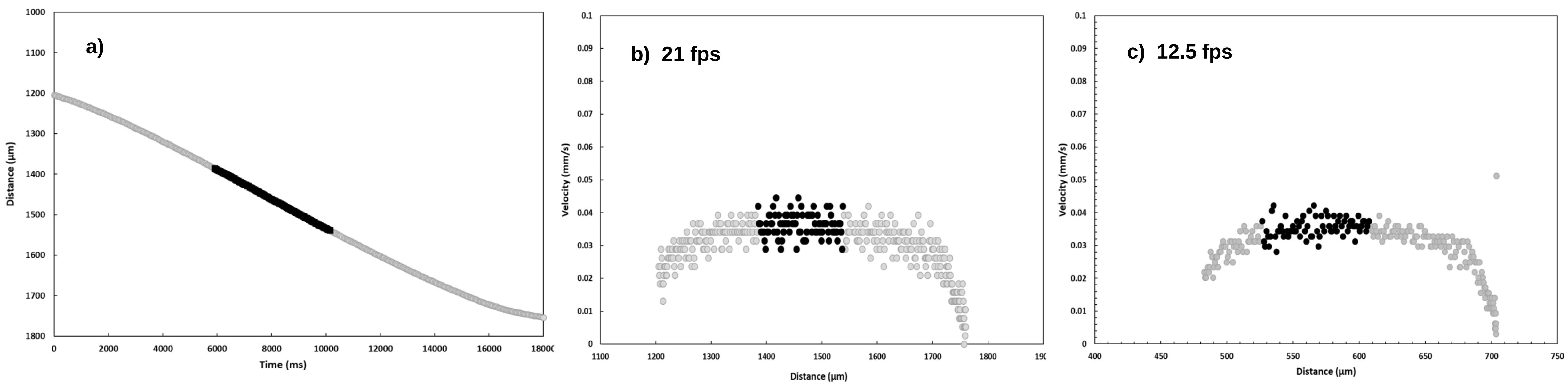

Figure S14 (a) The falling distance of the Pt sphere (diameter of 87 μm) for the run at 3.7 GPa and 1650 °C (APR16-PE7) as function of time. Falling velocity profiles are shown at b) 21 fps and c) 12.5 fps. The terminal velocity was calculated by averaging an interval of maximum velocities (black dots) achieved only at limited regions of falling distance (~1400–1550 μm distance).
